# Effect of AGG Interruptions on *FMR1* Maternal Transmissions

**DOI:** 10.3389/fmolb.2020.00135

**Published:** 2020-07-14

**Authors:** Olatz Villate, Nekane Ibarluzea, Hiart Maortua, Ana Belén de la Hoz, Laia Rodriguez-Revenga, Silvia Izquierdo-Álvarez, María Isabel Tejada

**Affiliations:** ^1^Biocruces Bizkaia Health Research Institute, Barakaldo, Spain; ^2^Genetics Service, Cruces University Hospital, Osakidetza Basque Health Service, Barakaldo, Spain; ^3^Centre for Biomedical Research on Rare Diseases (CIBERER), ISCIII, Madrid, Spain; ^4^Biochemistry and Molecular Genetics Department, Hospital Clinic, Barcelona, Spain; ^5^Institut d'Investigació Biomèdica August Pi i Sunyer IDIBAPS, Barcelona, Spain; ^6^Genetics Department of Clinical Biochemistry, Hospital Universitario Miguel Servet, Zaragoza, Spain

**Keywords:** *FMR1* gene, fragile X syndrome, CGG repeats, AGG interruptions, premutation, genetic counseling

## Abstract

There are four classes of CGG repeat alleles in the *FMR1* gene: normal alleles have up to 44 repeats; patients with Fragile X Syndrome have more than 200 repeats; those between 55 and 200 CGGs are considered *FMR1* premutation alleles, because they are associated with maternal expansions of the number of CGGs in the next generation and finally, alleles between 45 and 54 CGGs are called intermediate or gray zone alleles. In these last categories, the stability depends on the presence of AGG interruptions, which usually occurs between 9 and 10 CGGs. In this context, we have studied retrospectively 66 women with CGG repeats between 45 and 65, and their offspring. In total 87 transmissions were analyzed with triplet repeat primed PCR using AmplideX® FMR1 PCR (Asuragen, Austin, TX, USA) and we found that alleles with CGG repeats between 45 and 58 do not expand in the next generation except two cases with 56 repeats and 0 AGG interruptions. Furthermore, we have found four females with alleles with more than 59 CGG repeats and 2 AGG interruptions that do not expand either. Alleles from 56 CGG repeats without AGGs expand in all cases. In light of these results and those of the literature, we consider that the risk of unstable transmissions should be based on the presence or absence of AGG interruptions and not on the classical cutoffs which define each category of *FMR1* alleles. The application of these results in the genetic and reproductive counseling is essential and AGG interruptions should always be studied.

## Introduction

The *FMR1* gene is the gene responsible for Fragile X Syndrome (FXS) affecting ~1/3,717 to 1/8,918 Caucasian males (Crawford et al., 2001). FXS occurs when FMR1 is silenced by methylation or inactivation due to an abnormal expansion of a CGG trinucleotide (>200 repeats and called Full mutation, FM), located in the untranslated sequence at 5′, before the *FMR1* gene's first exon (Oberle et al., 1991; Verkerk et al., 1991; Yu et al., 1991).

It has been established that the normal CGG repeat number is below 45 and alleles in this repeat range are transmitted stably from generation to generation. Those alleles carrying between 45 and 200 CGG repeats are premutation (55–200 repeats, PM) or intermediate alleles (45–54 CGGs, IAs). The American College of Medical Genetics (ACMG) guidelines for defining these ranges are currently followed (Maddalena et al., 2001).

The term PM has been used since the discovery of the *FMR1* gene (Oberle et al., 1991) to reflect the fact that PM carriers do not generally have intellectual disability (ID) but their alleles are usually unstable, resulting in an expansion of the CGG repeats when transmitted by a female. It has been demonstrated that the risk of expansion is related to the number of CGG repeats, with smaller alleles being less likely to expand to a full mutation than larger ones (Rousseau et al., 1994; Nolin et al., 2003; Berkenstadt et al., 2007; Strom et al., 2007; Tejada et al., 2014) that is, the instability of PM alleles increases with the size of alleles. The smallest PM that has been reported to expand to a FM allele in one generation is 56 CGGs (Fernandez-Carvajal et al., 2009), later registered in our published cohort (Tejada et al., 2014).

The number of women carrying PM alleles is really high: it seems that as many as 1/130–260 females are carriers of a PM (Hagerman, 2008). In addition, these women could be at risk of developing several disorders associated with being a PM carrier: fragile X-associated premature ovarian insufficiency (FXPOI) (Sullivan et al., 2011), fragile X-associated tremor/ataxia syndrome (FXTAS) (Jacquemont et al., 2003), and the recently described fragile X-associated neuropsychiatric disorders (FXAND) (Hagerman et al., 2018). It has been estimated that about 13–26% of PM carriers women develop FXPOI (Wittenberger et al., 2007) and that up to 13% of women with PM could have FXTAS (Adams et al., 2007). In Spanish women with PM, we found similar frequencies to that previously reported for FXPOI (22.61%) (Merino et al., 2016), and also for FXTAS with frequencies from 3.27% (Merino et al., 2016) up to 16% (Rodriguez-Revenga et al., 2009).

There is also another range, that of the IAs or gray zone alleles (45–54 CGG), so called because these alleles may or may not be unstable (Nolin et al., 2003). In fact, we previously found that four mothers with 50–54 repeats expanded to a PM allele in the next generation (Tejada et al., 2014) and that 2% expanded to a FM in two generations (Madrigal et al., 2011). These alleles are very common in the population (0.8–3.6% with some geographical variability) (Madrigal et al., 2011) but their clinical relevance is not comparable to that of PM and it has been highly controversial. In previous studies in Spain, no evidence of an association between IA and behavioral or cognitive phenotypes was found (Madrigal et al., 2011) and, in relation to FXPOI and FXTAS, we found no association for alleles below 50 CGGs but the clinical implication of IA ≥50 CGGs was not clear and remains to be further elucidated (Alvarez-Mora et al., 2018).

Coming back to the expansion of a *FMR1* allele, today it is known that the risk of expansion depends not only on the number of CGGs but also on the presence of AGG interruptions (Eichler et al., 1994, 1996). In the general population, almost 95% of alleles have one or two AGG interruptions, and usually occur after 9–10 CGG repeats (Eichler et al., 1994, 1996). In the case of PM alleles, a combined international study has recently shown that there is a significant number of women with 55-90 CGG repeats that have one or more AGG interruptions, even up to five in some cases (Domniz et al., 2018). The biological function of these interruptions appears to be to stabilize the gene during transmission and to decrease the risk of DNA polymerase slippage during DNA replication (Yrigollen et al., 2012, 2014; Nolin et al., 2013, 2015, 2019). Presumably, the alleles without AGGs confer a high risk of unstable transmission (Dombrowski et al., 2002; Domniz et al., 2018) and on the contrary, the presence of AGG interruptions within the CGG repeat tract significantly increases genetics stability and reduces the risk of expansion to a greater number of CGGs (Yrigollen et al., 2012). Therefore, knowledge of the distribution of these interruptions in IAs and PM alleles is of great importance for genetic counseling.

Since our previous works were done only with data recorded with the size of CGGs, we undertook the retrospective study of the published cases, adding new cases of recent years in order to determine the exact sequence of the CGG repeat tract with AGGs in our population. The overall aim of this study is to shed light on this topic to improve genetic and reproductive counseling in women with IA and PM alleles and to suggest that the risk of expansion should be based on the study of AGG interruptions which have not been taken into account in the established ranges followed nowadays.

## Materials and Methods

### Study Design and Subjects

The design of the study was retrospective. Women with alleles between 45 and 65 CGG repeats were selected for the study. This range was selected based on the study of Nolin et al. (2013) where they found similar frequencies of unstable transmissions in the ranges of 60–64 CGG repeats and 65–69 CGG repeats and to understand the effect of AGGs in women with low PM (<65 CGG repeats) in order to provide a more accurate genetic counseling taking into account also recent studies on transmissions. In Cruces University Hospital these cases were taken anonymously from a database which includes the results of the *FMR1* alleles obtained between 1991 and 2018 in patients referred for Fragile X testing and their relatives in northern Spain. Collaborative hospitals from Barcelona and Zaragoza contributed with new cases for the study. In total, we studied 66 women (index cases) with CGG repeats between 45 and 65, and their children. Eighty-seven transmissions (47 males and 40 females) were analyzed. Informed consent approved by the clinical ethical committee was obtained in all cases prior to genetic testing.

### Molecular Analyses

Genomic DNA was extracted from peripheral blood. DNA samples were amplified with the AmplideX® FMR1 PCR commercial kit (Asuragen, Austin, TX, USA) to obtain the exact number of repeats of both CGGs and AGGs. Sequences were analyzed with the Gene Mapper™ Software and the formula was annotated for each sample.

## Results

### Index Cases

Table 1 shows the results of *FMR1* triplet repeat studies in the index cases (each woman with analyzed offspring) of the project indicating the CGG repeats and the AGG interruptions. The most common alleles found were those carrying 56 or 57 repeats with two AGG interruptions representing the 15% of our cases. We did not find any cases of 60 repeats in our study as an index case. For each case, the formula of the CGG repeats and AGG interruptions was recorded.

**Table 1:** Results of triplet studies indicating CGG repeats and AGG interruptions in index cases.

**Maternal CGGs**	**Maternal AGGs**	**Number of index cases**	**Total number of index cases**
45	1	1	3
	2	2	
46	1	2	4
	2	2	
47	1	1	4
	2	3	
48	0	1	5
	1	1	
	2	3	
49	1	1	3
	2	2	
50	1	2	4
	2	2	
51	2	2	2
52	2	3	3
53	2	4	4
54	1	1	2
	2	1	
55	1	2	4
	2	2	
56	0	2	7
	2	5	
57	1	1	6
	2	5	
58	1	1	2
	2	1	
59	0	2	4
	2	2	
61	0	1	1
62	0	1	3
	2	2	
63	0	1	2
	1	1	
64	0	1	2
	2	1	
65	2	1	1
		Total = 66	

*For each woman only the intermediate allele or the allele with premutation is represented*.

### Analysis of CGGs and AGGs Transmissions

For each index case, at least one generation was studied in order to analyze the transmission of the allele from 45 to 65 CGG repeats. In some cases, three generations of the same family could be studied. Also in several cases for each index case more than one child was studied, so the number of transmissions was greater than the number of index cases studied.

**Figure 1:**
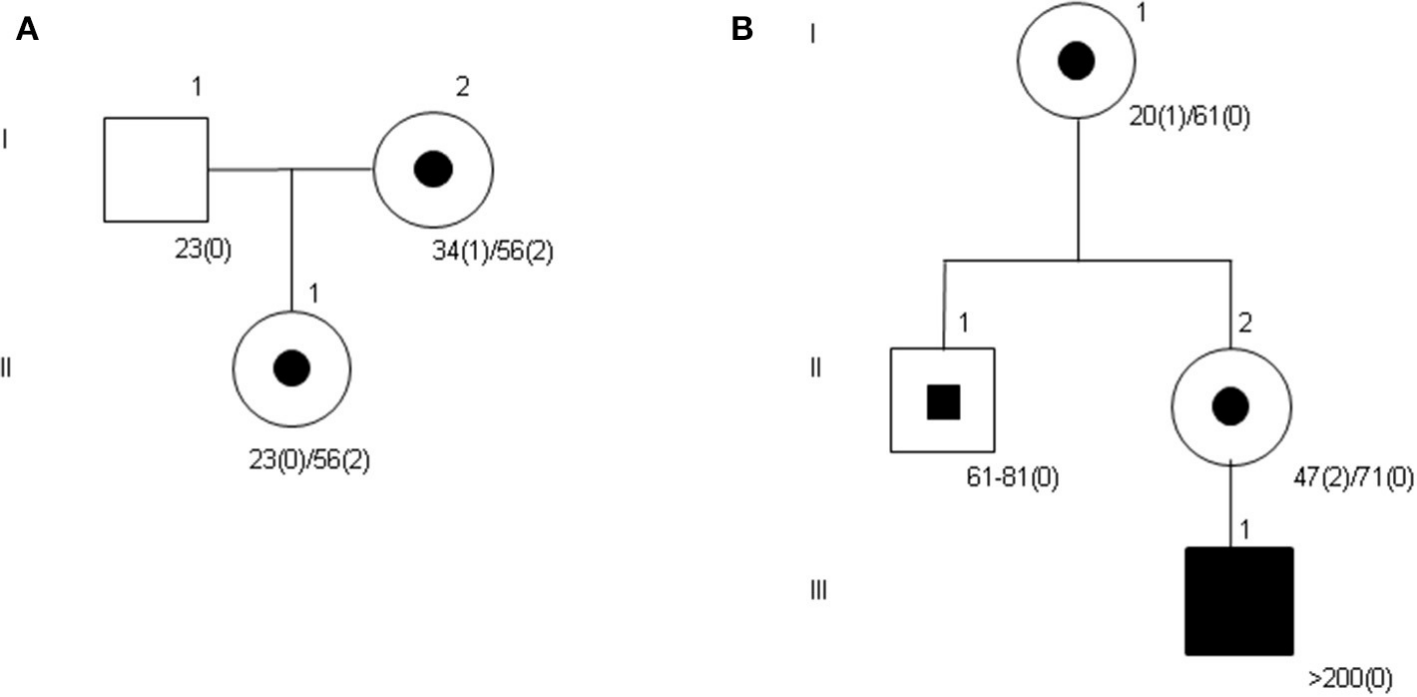
Examples of pedigrees of studied families showing the expansion of the CGG repeats. **(A)** The allele with 56 CGG repeats and two AGG interruptions is inherited by the daughter without expansion. **(B)** In the third generation, the patient presents with Fragile X Syndrome with an allele of more than 200 repeats. Subject II-1 presents a mosaicism in the number of CGG repeats.

Table 2 shows the results of the transmission of alleles based on AGGs interruptions. Alleles with CGG repeats between 45 and 58 do not expand in the next generation except two alleles with 56 repeats and no AGG interruptions which expand in one case to 62 repeats and in another case to 65 and 77 repeats in two different transmissions. Therefore, AGG interruptions confer stability to the alleles as is shown in Figure 1A. Furthermore, we have found four females with alleles with more than 59 CGG repeats and two AGG interruptions that do not expand either. Alleles from 56 CGG repeats without AGGs expand in all cases. Results of *FMR1* triplet repeat expansions over different generations within families where the index case presented no AGG interruptions in her PM allele are represented in Table 3. In all cases, the allele has expanded, leading in the third generation to a large expansion of CGG repeats and the appearance of FXS. In the second generation of these families where the index case does not present AGGs, in several cases CGG mosaicisms appear due to the instability of the allele. An example of the families where the allele has expanded due to the lack of AGG interruptions is represented in Figure 1B.

**Table 2:** Transmissions of the alleles between 45 and 65 CGG repeats.

**Maternal CGGs**	**Maternal AGGs**	**Number of index cases**	**CGGs (AGGs) in offspring**	**Total number of offspring**
45	1	1	45 (1)	1
	2	2	45 (2)	2
46	1	2	46 (1)	2
	2	2	46 (2)	2
47	1	1	47 (1)	1
	2	3	47 (2)	3
48	0	1	48 (0)	1
	1	1	48 (1)	1
	2	3	48 (2)	3
49	1	1	49 (1)	1
	2	2	49 (2)	2
50	1	2	48 (1)	2
	2	2	50 (2)	2
51	2	2	51 (2)	2
52	2	3	52 (2)	3
53	2	4	53 (2)	9
54	1	1	54 (1)	1
	2	1	54 (2)	1
55	1	2	55 (1)	2
	2	2	55 (2)	2
56	0	1	62 (0)	1
		1	65 (0)	1
			77 (0)	1
	2	5	56 (2)	6
57	1	1	57 (1)	2
	2	5	57 (2)	7
58	1	1	57 (1)	1
	2	1	58 (2)	1
59	0	1	68 (0)	1
			74–82 (0)	1
		1	82 (0)	1
	2	2	59 (2)	3
61	0	1	71 (0)	1
			61–81 (0)	1
62	0	1	>200	1
	2	1	62 (1)	2
	2	1	64 (2)	1
63	0	1	66 (0)	1
	1	1	125–150 (0)	1
64	0	1	80 (0)	1
	2	1	64 (2)	2
65	2	1	70(0)	1
			88(0)	1

*In the case of offspring with different formulas, they have been arranged on several lines. Alleles separated by a hyphen indicate the appearance of a mosaic in CGG triplets*.

**Table 3:** Results of triplet studies in different generations of families where the index case presented 0 AGG interruptions in her allele.

**1st generation CGGs (AGGs)**	**2nd generation CGGs (AGGs)**	**3rd generation CGGs (AGGs)**
56 (0)	62 (0)	>200 (0)
59 (0)	82 (0)	>200 (0)
59 (0)	74–82 (0)	>200 (0)
		>200 (0)
	68 (0)	N
61 (0)	71 (0)	>200 (0)
	61–81 (0)	N
64 (0)	80 (0)	>200 (0)

*N, indicates no offspring*.

## Discussion

Although the importance of AGG interruptions in maintaining *FMR1* repeat stability has been well-documented (Yrigollen et al., 2012; Nolin et al., 2013, 2015; Domniz et al., 2018), nowadays clinicians use in genetic and reproductive counseling the laboratory reports where the American College of Medical Genetics definition for the ranges of IA (45–54) and PM (55–200) (Maddalena et al., 2001) are followed. These guidelines do not include the composition of the sequence (i.e., presence or not of AGG interruptions). This raises a great anxiety in many women due to the high frequency of IAs and PM (Madrigal et al., 2011) in our population.

The number of AGGs has a substantial impact on the risk and the magnitude of repeat change from the mother to her offspring. In our study we have found that alleles within the range of 45–59 CGG repeats and two AGG interruptions do not expand in the next generation. Moreover, there are two cases of 64 CGG repeats and two AGG interruptions which do not expand to the next generation either. Nolin et al. (2013) analyzed the effect of maternal repeat size and number of AGGs on unstable transmissions and full mutation expansions and found that in the range of 45–49 CGG repeats and two AGGs there is a 5% of risk of unstable transmissions as well as in the range of 55–59 CGG repeats and two AGGs. These results show that the risk of expansion is the same in these particular ranges belonging to IA and low PM classifications. Domniz et al. (2018) analyzed PM carriers with 55–90 CGG repeats and found that the risk of unstable transmissions for alleles between 55 and 59 CGGs with two AGGs was a little bit higher (14.5% in the combined study) decreasing to 0% when they have more than two AGGs. They also showed that the risk increases to 50% in the range of 60–64 CGGs with two or more AGGs. In our study including less cases, we have also found that alleles below 60 CGG repeats with two AGGs do not expand in the next generation but above 60 CGGs we observe a small increase in the risk of unstable transmissions (two cases with 62 CGGs and two AGGs in Table 2; in one case there is a loss of one AGG and in the other case there is an expansion of two CGGs). Furthermore, in the previous Spanish study on IAs (Madrigal et al., 2011), the analysis of 100 transmissions of IAs showed that 95% of these alleles were stable, only 3% expanded within the same range and 2% expanded to a FM in two generations; these two cases had lost the AGG interruptions. In the present study and in the range of IAs, we have only found one case without AGGs (48 CGGs) that did not expand in the next generation (Table 2), but all the alleles from 56 CGG repeats without AGG interruptions, expanded in the next generation giving a FXS in the third generation. All FM expansions were transmitted from alleles lacking AGGs (Table 3 and Figure 1B). These results show once again that AGG interruptions give stability to the allele as seen in other recent studies (Nolin et al., 2013, 2015, 2019; Yrigollen et al., 2014).

The discovery of AGG interruptions in IA or low PM has a great application in the genetic and reproductive counseling of carriers. Although we have a limited number of cases, with this study and the results of other recent studies, we suggest that the risk of expansion should be personalized and based on the study of AGG interruptions which have not been taken into account in the established ranges followed nowadays. In a previous study of CGG transmissions where AGG interruptions were not analyzed (Tejada et al., 2014) it was concluded that women with 55–59 CGG presented a risk of expansion to FM of 6.4%. And in this study we observe, for example, five index cases with 57 CGG repeats and two AGGs which do not expand in the seven transmissions analyzed. Finally, PM alleles may lose AGGs and CGGs through contractions and become normal, intermediate, or smaller premutation alleles (Nolin et al., 2019) so follow-up of women with a very low number of CGG repeats and no AGG interruptions should be recommended, despite the low risk of expansion in the next generation, like the case we have with 48 CGGs.

In conclusion, we strongly recommend that clinical laboratories study the total number of CGG repeats with the number of AGG interruptions as it was already said in previous studies (Yrigollen et al., 2012; Domniz et al., 2018) but unfortunately it is not a routine in many laboratories. In genetic counseling, it will be very important to convey this more accurate risk information to women carriers of alleles with 45–65 CGG repeats regarding their risk of expansion or not of their CGG repeats in the *FMR1* gene. This will serve to reassure women with very low risk of expansion even if they are classified as PM carrier according to the classic definition (i.e., <59 CGGs with two AGGs), and to follow-up those that have higher risk including those with IAs and no AGGs.

## Data Availability Statement

All datasets presented in this study are included in the article.

## Ethics Statement

Informed consent approved by the clinical ethical committee was obtained in all cases prior to genetic testing and it includes the permission of using the data for statistical analyses.

## Author Contributions

OV and MT designed the study. OV wrote the manuscript. OV, NI, HM, AH, LR-R, and SI-Á collected data and performed the analyses. MT supervised the entire work. All authors revised the manuscript critically, approved the final manuscript as submitted and agreed to be accountable for all aspects of the work All authors contributed to the article and approved the submitted version.

## Conflict of Interest

The authors declare that the research was conducted in the absence of any commercial or financial relationships that could be construed as a potential conflict of interest.

## References

[B1] Adams J. S. Adams P. E. Nguyen D. Brunberg J. A. Tassone F. Zhang W.. (2007). Volumetric brain changes in females with fragile X-associated tremor/ataxia syndrome (FXTAS). Neurology 69, 851–859. 10.1212/01.wnl.0000269781.10417.7b 17724287

[B2] Alvarez-Mora M. I. Madrigal I. Martinez F. Tejada M. I. Izquierdo-Alvarez S. Sanchez-Villar de Saz P.. (2018). Clinical implication of FMR1 intermediate alleles in a Spanish population. Clin. Genet. 94, 153–158. 10.1111/cge.13257 29604051

[B3] Berkenstadt M. Ries-Levavi L. Cuckle H. Peleg L. Barkai G. (2007). Preconceptional and prenatal screening for fragile X syndrome: experience with 40,000 tests. Prenat. Diagn. 27, 991–994. 10.1002/pd.1815 17705235

[B4] Crawford D. C. Acuña J. M. Sherman S. L. (2001). FMR1 and the fragile X syndrome: human genome epidemiology review. Genet. Med 3, 359–371. 10.1097/00125817-200109000-00006 11545690 PMC4493892

[B5] Dombrowski C. Levesque S. Morel M. L. Rouillard P. Morgan K. Rousseau F. (2002). Premutation and intermediate-size FMR1 alleles in 10572 males from the general population: loss of an AGG interruption is a late event in the generation of fragile X syndrome alleles. Hum. Mol. Genet. 11, 371–378. 10.1093/hmg/11.4.371 11854169

[B6] Domniz N. Ries-Levavi L. Cohen Y. Marom-Haham L. Berkenstadt M. Pras E.. (2018). Absence of AGG interruptions is a risk factor for full mutation expansion among israeli FMR1 premutation carriers. Front. Genet. 9:606. 10.3389/fgene.2018.00606 30619448 PMC6300753

[B7] Eichler E. E. Holden J. Popovich B. W. Reiss A. L. Snow K. Thibodeau S. N.. (1994). Length of uninterrupted CGG repeats determines instability in the FMR1 gene. Nat. Genet. 8, 88–94. 10.1038/ng0994-88 7987398

[B8] Eichler E. E. Macpherson J. N. Murray A. Jacobs P. A. Chakravarti A. Nelson D. L. (1996). Haplotype and intersperson analysis of the FMR1 CGG repeat indentifies two different mutational pathways for the origin of the fragile X syndrome. Hum. Molec. Genet. 5, 319–330. 10.1093/hmg/5.3.319 8852655

[B9] Fernandez-Carvajal I. Lopez Posadas B. Pan R. Raske C. Hagerman P. J. Tassone F. (2009). Expansion of an FMR1 grey-zone allele to a full mutation in two generations. J. Mol. Diagn. 11, 306–310. 10.2353/jmoldx.2009.080174 19525339 PMC2710706

[B10] Hagerman P. J. (2008). The fragile X prevalence paradox. J. Med. Genet. 45, 498–499. 10.1136/jmg.2008.059055 18413371 PMC2728763

[B11] Hagerman R. J. Protic D. Rajaratnam A. Salcedo-Arellano M. J. Aydin E. Y. Schneider A. (2018). Fragile X-associated neuropsychiatric disorders (FXAND). Front. Psychiatry 13:564 10.3389/fpsyt.2018.00564 PMC6243096 30483160

[B12] Jacquemont S. Hagerman R. J. Leehey M. Grigsby J. Zhang L. Brunberg J. A.. (2003). Fragile X premutation tremor/ataxia syndrome: molecular, clinical, and neuroimaging correlates. Am. J. Hum. Genet. 72, 869–878. 10.1086/374321 12638084 PMC1180350

[B13] Maddalena A. Richards C. S. McGinniss M. J. Brothman A. Desnick R. J. Grier R. E.. (2001). Technical standards and guidelines for fragile X: the first of a series of disease-specific supplements to the standards and guidelines for clinical genetics laboratories of the american college of medical genetics. quality assurance subcommittee of the laboratory practice committee. Genet. Med. 3, 200–205. 10.1097/00125817-200105000-00010 11388762 PMC3110344

[B14] Madrigal I. Xunclà M. Tejada M. I. Martínez F. Fernández-Carvajal I. Pérez-Jurado L. A.. (2011). Intermediate FMR1 alleles and cognitive and/or behavioural phenotypes. Eur. J. Hum. Genet. 19, 921–923. 10.1038/ejhg.2011.41 21427756 PMC3172924

[B15] Merino S. Ibarluzea N. Maortua H. Prieto B. Rouco I. López-Aríztegui M. A.. (2016). Associated clinical disorders diagnosed by medical specialists in 188 FMR1 premutation carriers found in the last 25 years in the spanish basque country: a retrospective study. Genes 7:E90. 10.3390/genes7100090 27775646 PMC5083929

[B16] Nolin S. L. Brown W. T. Glicksman A. Houck G. E. Jr. Gargano A. D. Sullivan A.. (2003). Expansion of the fragile X CGG repeat in females with premutation or intermediate alleles. Am. J. Hum. Genet.72, 454–464. 10.1086/367713 12529854 PMC379237

[B17] Nolin S. L. Glicksman A. Ersalesi N. Dobkin C. Brown W. T. Cao R.. (2015). Fragile X full mutation expansions are inhibited by one or more AGG interruptions in premutation carriers. Genet. Med. 17, 358–364. 10.1038/gim.2014.106 25210937

[B18] Nolin S. L. Glicksman A. Tortora N. Allen E. Macpherson J. Mila M.. (2019). Expansions and contractions of the FMR1 CGG Repeat in 5,508 transmissions of normal, intermediate, and premutation alleles. Am. J. Med. Genet. A 179, 1148–1156. 10.1002/ajmg.a.61165 31050164 PMC6619443

[B19] Nolin S. L. Sah S. Glicksman A. Sherman S. L. Allen E. Berry-Kravis E.. (2013). Fragile X AGG analysis provides new risk predictions for 45-69 repeat alleles. Am. J. Med. Genet. A 161A, 771–778. 10.1002/ajmg.a.35833 23444167 PMC4396070

[B20] Oberle I. Rousseau F. Heitz D. Kretz C. Devys D. Hanauer A.. (1991). Instability of a 550-base pair DNA segment and abnormal methylation in fragile X syndrome. Science 252, 1097–1102. 10.1126/science.252.5009.1097 2031184

[B21] Rodriguez-Revenga L. Madrigal I. Pagonabarraga J. Xuncl,à M. Badenas C. Kulisevsky J.. (2009). Penetrance of FMR1 premutation associated pathologies in fragile X syndrome families. Eur. J. Hum. Genet. 17, 1359–1362. 10.1038/ejhg.2009.51 19367323 PMC2986640

[B22] Rousseau F. Heitz D. Tarleton J. MacPherson J. Malmgren H. Dahl N.. (1994). A multicenter study on genotype-phenotype correlations in the fragile X syndrome, using direct diagnosis with probe StB12.3: the first 2,253 cases. Am. J. Hum. Genet. 55, 225–237. 8037202 PMC1918361

[B23] Strom C. M. Crossley B. Redman J. B. Buller A. Quan F. Peng M.. (2007). Molecular testing for fragile X syndrome: lessons learned from 119,232 tests performed in a clinical laboratory. Genet. Med. 9, 46–51. 10.1097/GIM.0b013e31802d833c 17224689

[B24] Sullivan S. D. Welt C. Sherman S. (2011). FMR1 and the continuum of primary ovarian insufficiency. Semin. Reprod. Med. 29, 299–307. 10.1055/s-0031-1280915 21969264

[B25] Tejada M. I. Glover G. Martínez F. Guitart M. de Diego-Otero Y. Fernández-Carvajal I.. (2014). Molecular testing for fragile X: analysis of 5062 tests from 1105 fragile X families–performed in 12 clinical laboratories in Spain. Biomed Res. Int. 2014:195793. 10.1155/2014/195793 24987673 PMC4058505

[B26] Verkerk A. J. Pieretti M. Sutcliffe J. S. Fu Y. H. Kuhl D. P. Pizzuti A.. (1991). Identification of a gene (FMR-1) containing a CGG repeat coincident with a breakpoint cluster region exhibiting length variation in fragile X syndrome. Cell 65, 905–914. 10.1016/0092-8674(91)90397-H 1710175

[B27] Wittenberger M. D. Hagerman R. J. Sherman S. L. McConkie-Rosell A. Welt C. K. Rebar R. W.. (2007). The FMR1 premutation and reproduction. Fertil. Steril. 87, 456–465. 10.1016/j.fertnstert.2006.09.004 17074338

[B28] Yrigollen C. M. Durbin-Johnson B. Gane L. Nelson D. L. Hagerman R. Hagerman P. J.. (2012). AGG interruptions within the maternal FMR1 gene reduce the risk of offspring with fragile X syndrome. Genet. Med. 14, 729–736. 10.1038/gim.2012.34 22498846 PMC3990283

[B29] Yrigollen C. M. Martorell L. Durbin-Johnson B. Naudo M. Genoves J. Murgia A.. (2014). AGG interruptions and maternal age affect FMR1 CGG repeat allele stability during transmission. J. Neurodev. Disord. 6:24. 10.1186/1866-1955-6-24 25110527 PMC4126815

[B30] Yu S. Pritchard M. Kremer E. Lynch M. Nancarrow J. Baker E.. (1991). Fragile X genotype characterized by an unstable region of DNA. Science 252, 1179–1181. 10.1126/science.252.5009.1179 2031189

